# Finnish healthcare professionals' attitudes towards robots: Reflections on a population sample

**DOI:** 10.1002/nop2.138

**Published:** 2018-03-23

**Authors:** Tuuli Turja, Lina Van Aerschot, Tuomo Särkikoski, Atte Oksanen

**Affiliations:** ^1^ Faculty of Social Sciences University of Tampere Tampere Finland

**Keywords:** attitudes, healthcare worker, quantitative approaches, technology

## Abstract

**Aim:**

To answer the question: ‘How prepared healthcare professionals are to take robots as their assistants in terms of experience and acceptance?’

**Background:**

The ageing population, increasing care needs and shortage of healthcare professionals pose major challenges in Western societies. Special service robots designed for care tasks have been introduced as one solution to these problems.

**Design:**

A correlative design

**Methods:**

Eurobarometer data (*N *=* *969) and survey data of nurses and other healthcare professionals (*N* = 3800) were used to assess the relationship between robot acceptance and experiences with robots while controlling for the respondents’ age, gender, occupational status and managerial experience.

**Results:**

Healthcare professionals had less experience with robots and more negative attitudes towards them than the general population. However, in healthcare, robot assistance was welcomed for certain tasks. These regarded, for example, heavy lifting and logistics. Previous experiences with robots were consistently correlated with robot acceptance.

## 1 INTRODUCTION

The ageing of the population, increasing care needs and probable shortage of healthcare workers pose major societal challenges in many Western societies. New technologies and novel generations of service robots are expected to open opportunities to develop renewed ways of providing care and, moreover, enable significant reforms to service provision and care work (Baer, Tilliette, Jeleff, Ozguler, & Loeb, 2014; Decker et al., 2011). Robots are expected to alleviate the shortage of care professionals (Zsiga et al., 2013) and to increase the autonomy of elderly people (Sorell & Draper, 2014).

Robots are programmable mechatronic devices that can move and perform tasks in their environment. Care robots, referring to service robots in a care context, are devices that conduct tasks autonomously, semi‐autonomously or through teleoperation (Goeldner, Herstatt, & Tietze, 2015). A care robot may be integrated into care practices and either assist healthcare personnel or work directly with care receivers. Care robots are foreseen to be used as, for example, assistants that help with daily tasks like toileting, dressing and getting from a bed to a wheelchair (Pino, Boulay, Jouen, & Rigaud, 2015; Shin & Choo, 2011; Sparrow & Sparrow, 2006), increasing the autonomy of disabled persons or frail older people. Second, robots can be used in care context as they are already used in households doing basic routine tasks like cleaning or lawn mowing (Katz & Halpern, 2014). Third, monitoring robots can keep track and register health‐ and safety‐related factors and call for help when needed (Sharkey & Sharkey, 2012). Fourth, social robots are designed for emotional, cognitive and physical rehabilitation for people who, for example, suffer from dementia (Sharkey & Sharkey, 2012; Sparrow & Sparrow, 2006).

A major concern regarding the introduction of novel care technology is whether it affects the care recipients in a positive or negative manner (Borenstein & Pearson, 2010). People are worried that robots will replace workers in healthcare and hence endanger the quality, ethical principles and current standards of care work (Beedholm, Frederiksen, Skovsgaard Frederiksen, & Lomborg, 2015; Hofmann, 2013; Sharkey, 2014; Vallor, 2011). Robots arguably “pose a threat to the holistic care” (van Wynsberghe, 2013, p. 427). Signs of these concerns are also visible in public opinion. Only about 4% of Europeans think that robots are suitable for care of children, elderly and the disabled (Special Eurobarometer 382, 2012).

### 1.1 Background

In general, men, younger adults and those with higher education are more prone to accept robots (de Graaf & Ben Allouch, 2013). Positive attitudes towards robots also consistently correlate with the amount of experiences with robotic devices (Heerink, 2011; Louie, McColl, & Nejat, 2014; Nomura, Kanda, & Suzuki, 2006). However, studies on healthcare professionals’ attitudes towards robots are rare. Prior findings imply that nurses appreciate robots as assistive tools and monitoring devices, but not for tasks that require social interaction (Alaiad & Zhou, 2014; Jenkins & Draper, 2015). In Beedholm et al. (2015) study, managers of an elderly centre had more positive views of a bathtub robot compared with other staff and residents. Apart from the ethical discussion, managers appreciated the image‐elevating advantages that robotization would bring to their unit.

Remotely operated robots are more positively appraised than autonomous robots (Savela, Turja, & Oksanen, 2017). Healthcare professionals see exceptional potential in telepresence robots used in telecare (Koceski & Koceska, 2016; Kristoffersson, Coradeschi, Loutfi, & Severinson‐Eklundh, 2011). Via telepresence, healthcare professionals can visit patients from a remote location. Similarly, telepresence can be used, for example, as mobile video communication devices between home care clients and their relatives. Telepresence offers an important and interesting prospect of reorganizing and prioritizing tasks in care work. At the moment, nursing staff is concerned about resource shortages as well as administrative tasks and travelling taking time from actual care work (Ausserhofer et al., 2014; Ball, Murrells, Rafferty, Morrow, & Griffiths, 2014; Kodama & Fukahori, 2017; Menon, 2015; Trydegård, 2012).

Care for older people has its own specific challenges, such as time pressure in home care (Andersen & Westgaard, 2013) and a heightened risk of clients’ or their relatives’ violent behaviour (Banerjee et al., 2012). In Finland, a majority (70%) of practical nurses describe their work as too laborious and are considering a change in occupation (Erkkilä, Simberg, & Hyvärinen, 2016). Moreover, among nursing students, care for older patients is not perceived as a desirable field of work (Koskinen, Salminen, Stolt, & Leino‐Kilpi, 2014).

Beyond the aspect of reorganizing work, introducing care robots often aims at cost savings (Qureshi & Syed, 2014). Athough efforts have been made to apply econometric criteria (Preston, 1993) or management theories like the *theory of constraints* (Groop, 2012) to productivity gains in care services, it has been emphasized that care is essentially different from the production of other goods and services. The values of nursing require practitioners to make the care of people their first concern, focusing on respectfulness, compassion, trustworthiness, partnership, competence and safety (NMC, 2015; Scammell, Tait, White, & Tait, 2017). However, the care‐receivers’ rights to autonomy, choice and control have also been increasingly emphasized. People with disabilities and care needs should be able to make choices regarding the assistance and help they receive (Fine, 2007, p. 92–95; Kröger, 2009). Hence, care robots are seen as both posing a risk to good‐quality care and bringing positive chances for increasing the autonomy of care receivers.

According to Aimee van Wynsberghe (2013), robots should be designed to support and promote the fundamental values of care. It is possible to decide whether care robots are suitable and acceptable for different care tasks when we can tell which values are involved in those activities and whether these values and the initial points of the care tasks are endangered or fostered (Santoni de Sio & van Wynsberghe, 2016). The nature of activities approach distinguishes between goal‐directed activities and practice‐oriented activities. The point of goal‐directed activities is to reach an end that is external to the activity, whereas the main point of practice‐oriented activities is the performance of the activity itself (Santoni de Sio & van Wynsberghe, 2016). Thinking about care work more concretely, the different tasks can be divided into direct patient care, indirect patient care and other activities, including, for example, documentation, administration and planning medication (Ballermann, Shaw, Mayers, Gibney, & Westbrook, 2011).

As care robots are not yet commonly used by healthcare professionals, we need theoretical tools for analysing the suitability of robots for care work. In this study, we examined healthcare professionals’ experiences with robots and how these experiences associate with the general view of robots (GVR) or robot acceptance at work (RAW). We compared the GVR and RAW of healthcare professionals using a survey data of Finnish healthcare professionals to the attitudes of the general population, using Eurobarometer data for Finland.

In addition, we examined the tasks for which healthcare professionals consider the idea of robot assistance to be most agreeable and used the nature of activities theory to gain an understanding of the future prospects of introducing robots to care work. Our first hypothesis is that the respondents approve robot assistance for indirect patient care tasks rather than for direct and practice‐oriented tasks. This includes the idea of indirect patient care being aside the actual care work (Ausserhofer et al., 2014; Ball et al., 2014; Menon, 2015). Furthermore, because previous studies have shown that nurses perceive physical demands as one of the main challenges in their work (Erkkilä et al., 2016; Kodama & Fukahori, 2017; Trydegård, 2012), our second hypothesis was that the respondents are more approving towards robots that assist in physically burdening care tasks.

## 2 METHOD

### 2.1 Design

Quantitative, correlative study was conducted via three questionnaire data sets. Two identical surveys for Finnish healthcare professionals were filled out online. Questionnaire for Eurobarometer population sample, then, was executed as structured, face‐to‐face interviews.

### 2.2 Data

Survey data of healthcare professionals were collected from October to November 2016. The first sample was randomly selected from the members of the Finnish Union of Practical Nurses, who were currently working with the elders (*N = *2218). Every other individual in the population was selected with an equal likelihood of selection. The participants were aged 17–68 (mean = 45.5; *SD* 12.1) and 89.8% were female. The second sample was collected from the Union of Health and Social Care Professionals in Finland. The random sampling included every nurse (practical or registered) and physiotherapist currently working at elderly and homecare services as well as every third randomly selected nurse and physiotherapist working at a health centre or a hospital. The second sample consisted of mostly female (89.0%) nurses (*N = *1701) and physiotherapists (*N = *81) aged 19–70 (mean = 47.5; *SD* 10.4).

The questionnaire included multiple choice questions about educational and occupational background, experiences with assistive tools in healthcare and attitudes towards robots. Respondents who completed the first page of sociodemographic information and a question concerning interest in technology were included in the final and combined sample (*N* = 3800). Table 1 summarizes the background characteristics of the respondents and compares them to the population statistics of Finnish nurses (Ailasmaa, 2014).

**Table 1:** Occupational, gender and age distributions: Comparisons with the population

	Finnish population	Care worker sample
	2013	2016
Distribution between nurses		
Practical nurses	64.7%	64.9%
Registered nurses	35.3%	35.1%
Females		
Practical nurses	90.4%	95.1%
Registered nurses	92.1%	94.8%
Head nurses	93.3%	95.8%
Physiotherapists	81.2%	91.3%
Age mean		
Practical nurses	43.0	45.3
Registered nurses	43.3	47.5
Head nurses	50.7	53.6
Physiotherapists	42.6	47.1

In addition to the healthcare professional data, we used a Finnish population sample retrieved from the Eurobarometer statistics of 2014 (*N =* 969). The European Commission‐funded Eurobarometer polls monitor EU citizens’ social and political views and account for phenomena such as robotization. The Eurobarometer respondents were aged 15–91 (mean = 48.3; *SD* 19.06), with 51.3% being female.

### 2.3 Measures

**Figure 1:**
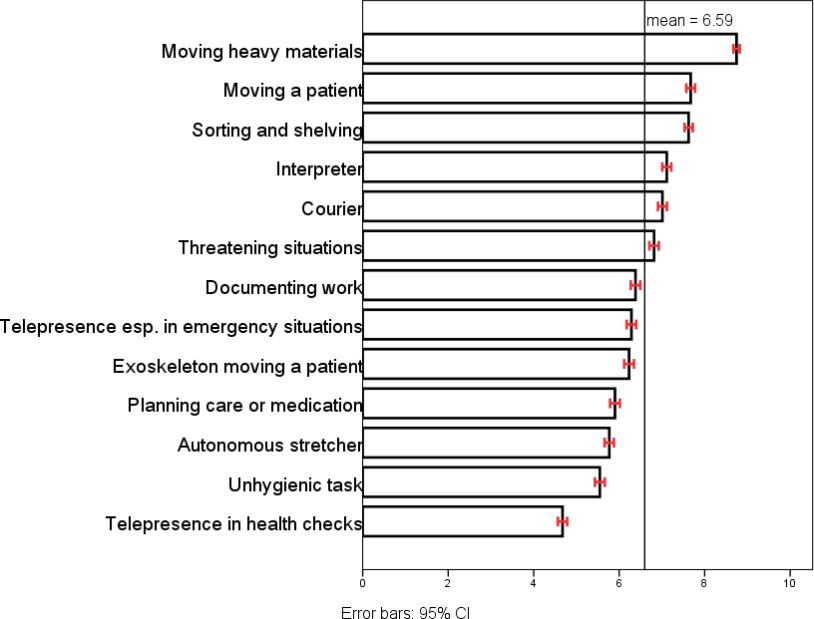
Average assessments for “How comfortable would you feel about a robot assisting you with a care work‐related task?

Attitudes towards robots were covered by two variables: the general view of robots (GWR) and robot acceptance at work (RAW). GVR was measured with the question: “Generally speaking, do you have a ‘very positive’ (4); ‘fairly positive’ (3); ‘fairly negative’ (2); or ‘very negative’ (1) view of robots?” This question was presented identically in the Eurobarometer and the surveys for the healthcare professionals. In the Eurobarometer questionnaire, RAW was measured with a question on how the respondent felt about “having a robot assist them at work (e.g., in manufacturing).” The scale for the answers ranged from 1 (totally uncomfortable) to 10 (totally comfortable) in the healthcare professionals’ surveys, RAW was summed from 13 questions concerning robots assisting in different care work scenarios (excluding robotic surgery). The scale for the answers ranged from 1 (totally uncomfortable)–10 (totally comfortable). The composite variable (range 13–130) was returned to its original scale from 1–10 (α 0.933). Figure 1 shows the means for all 13 scenarios and the full questions are presented in Appendix S1.

The definition of robots used to prime the questions on both the Eurobarometer and healthcare questionnaires was as follows: “Robot is defined as a machine which can assist humans in everyday tasks without constant guidance or instruction, e.g., as a kind of co‐worker helping on the factory floor or as a robot cleaner, or in activities which may be dangerous for humans, like search and rescue in disasters. Robots can come in many shapes or sizes and some may be of human appearance. Traditional kitchen appliances, such as a blender or a coffee maker, are not considered as robots” (Eurobarometer questionnaire 2014).

Experiences with robots were inquired about by asking the respondents whether they possessed concrete experiences with robots in different contexts: home, work and elsewhere. In addition, the healthcare professionals’ experiences with robots at work were obtained by asking whether there had been experiences in care work or other work. A composite variable formed a scale from 0–3 in the Eurobarometer sample and a scale from 0–4 in the healthcare professional samples. Zero, indicating no experience anywhere, was the mode in every data set.

In the questionnaires, the participants were asked about their age, gender, occupational status and managerial experience. Occupational status at the time of the study was dichotomized as employed (1) or not (0). Among the respondents of the population sample, 38.1% and 92.2% of the healthcare professional sample were employed at the time of the study. The healthcare professional surveys included a question about having managerial experience (1) or not (0). 17.7% reported having managerial experience. In the population sample, 18.4% of respondents described themselves as managers or other white‐collar employees.

### 2.4 Statistical analysis

The descriptive findings are presented as percentages, confidence intervals, means (*M*) and standard deviations (*SD*), along with Pearson correlations (*r*), *z*‐tests for an observed proportion and variance analysis (*F*) for group comparisons. Error bars used in the figure indicate statistical significance between means.

The relationship between attitudes towards robots and different experiences with robots, as well as some important background variables, was tested using multiple ordinary least squares (OLS) regression analysis. Variables were entered together into the analysis. This predictive analysis was applied separately for the population data and for the aggregate data of the two healthcare professionals’ samples. In total, four regression models are presented: GVR and RAW in the population data and GVR and RAW in the healthcare professional data. The results are reported as both unstandardized (average change in the dependent variable) and standardized (comparable effect size) regression coefficients.

## 3 RESULTS

Experiences with robots were more frequent among the population (16.2%) than among the healthcare professionals (11.9%; *z* = 4.13, *p* < .0001). The differences between different healthcare occupations were not statistically significant, as defined by the 95% confidence intervals. Table 2 shows specific percentages of usage context and profession.

**Table 2:** Percentages of robot use by context and sample. Care worker sample subgrouped by occupation

Have used a robot %	Population sample (*N *=* *969)	Practical nurses (*N *=* *1914)	Registered nurses (*N *=* *1032)	Head nurses/other managers (*N *=* *129)	Physiotherapists/rehabilitation workers (*N *=* *86)	Other healthcare workers (*N *=* *238)^c^
At home	3.7	6.3	7.5	10.9	9.3	6.3
At care work		2.9	3.0	3.9	2.3	1.3
At other work^a^	9.8	2.1	0.5	2.3	1.2	2.1
Elsewhere^b^	3.2	1.5	1.6	1.6	5.8	3.8
Anywhere	16.2	11.2	12.2	15.5	16.3	12.7

a This indicates any work context regarding the population sample.

b Such as fairs, info service, traffic and a friend's house.

c Such as public health nurse, medical receptionist.

Regarding GVR, the attitudes of the general population were more positive (mean = 2.85; *SD* .71) compared with the healthcare professionals (mean = 2.57; *SD* .71). The difference between the population sample and the aggregate healthcare professional sample was statistically significant based on the 95% confidence interval. Those Eurobarometer respondents, who were currently employed, had even more positive view on robots in general (mean = 2.94; *SD* .68).

Among the healthcare professionals, head nurses and other managers had the most positive views of robots (mean = 2.84; *SD* .643), followed by physiotherapists and rehabilitation workers (mean = 2.72; *SD* .680), registered nurses (mean = 2.70; *SD* .677) and practical nurses (mean = 2.46; *SD* .725) [*F*(4) = 25.35; *p* < .001]. Dunnett's T3 test revealed that the significant difference was between practical nurses and all other occupational groups.

In an opposite vein, a lower average level of RAW was found among the population (mean = 5.99; *SD* 2.95) compared with the healthcare professionals (mean = 6.57; *SD* 2.19). Among the healthcare professionals, head nurses had the highest acceptance of robots at work (mean = 7.42; *SD* 1.78), followed by registered nurses (mean = 6.95; *SD* 2.05), physiotherapists/rehabilitation workers (mean = 6.80; *SD* 1.83) and practical nurses (mean = 6.26; *SD* 2.24).

Figure 1 shows the average assessment of robot assistance for each care work task. Tasks involving heavy lifting received the most positive assessments and, hence, heightened the total RAW the most in the data of healthcare professionals. Correlations between healthcare professionals’ GVR, RAW, gender, age, managerial experience and total experiences of robots are presented in Appendix S2.

When the analysis was limited to the respondents who had personal experience with robots, we found higher average acceptance and less variance in assessments of GVR and RAW. This was further analysed by OLS regression models to assess the relationship between robot acceptance and the respondents’ experiences of robots while controlling for age, gender, employment status and managerial experience. The results from the population data are shown in Tables 3 and 4 and the results from the healthcare professionals are shown in Tables 5 and 6.

**Table 3:** General view on robots, regression analysis for the population data (*N *=* *969)

GVR (1–4), Population	Unstandardized		Standardized	
	*B*	*SE*	β	*p*
(Constant)	3.413	0.105		.000
Female	−0.230	0.044	−0.163	.000
Age	−0.005	0.001	−0.126	.000
Employed	−0.087	0.060	−0.060	.148
Manager/white‐collar	0.230	0.070	0.127	.001
Robots used at home	0.404	0.115	0.110	.000
Robots used at work	0.199	0.074	0.085	.007
Robots used elsewhere	0.115	0.126	0.029	.363

**Table 4:** Robot acceptance at work, regression analysis for the population data (*N *=* *969)

RAW (1–10), Population	Unstandardized		Standardized	
	*B*	*SE*	β	*p*
(Constant)	9.127	0.431		.000
Female	−1.016	0.184	−0.172	.000
Age	−0.029	0.005	−0.190	.000
Employed	−0.823	0.245	−0.136	.001
Manager/white‐collar	0.884	0.291	0.117	.002
Robots used at home	0.346	0.481	0.022	.472
Robots used at work	1.399	0.303	0.144	.000
Robots used elsewhere	0.623	0.520	0.037	.231

**Table 5:** General view on robots, regression analysis for the care worker data (*N *=* *3399)

GVR (1–4), care workers	Unstandardized		Standardized	
	*B*	*SE*	β	*p*
(Constant)	2.352	0.069		.000
Female	−0.232	0.060	−0.074	.000
Age	0.002	0.001	0.037	.057
Employed	0.031	0.051	0.012	.538
Managerial experience	0.164	0.035	0.090	.000
Robots used at home	0.325	0.051	0.121	.000
Robots used at healthcare	0.142	0.077	0.035	.066
Robots used at other work	0.289	0.102	0.054	.004
Robots used elsewhere	0.196	0.094	0.039	.039

**Table 6:** Robot acceptance at work, regression analysis for the care worker data (*N *=* *3399)

RAW (1–10), care workers	Unstandardized		Standardized	
	*B*	*SE*	β	*p*
(Constant)	5.827	0.201		.000
Female	0.071	0.179	0.007	.692
Age	0.011	0.004	0.057	.002
Employed	0.067	0.148	0.008	.650
Managerial experience	0.453	0.103	0.080	.000
Robots used at home	0.863	0.154	0.101	.000
Robots used at healthcare	0.041	0.231	0.003	.860
Robots used at other work	0.632	0.315	0.036	.045
Robots used elsewhere	0.360	0.294	0.022	.220

In our data, RAW was assessed the highest among those with managerial experience and those who had used robots in their work. Among the population, higher acceptance was also associated with male gender, not working at the time of the study and younger age. Among healthcare professionals, younger age, on the contrary, predicted a lower level of RAW. GVR was consistently the highest among men, those who had managerial experience and those who had used robots in their work.

Personal experiences with robots in different contexts were systematically associated with higher robot acceptance in all four models. In the population sample, those who had used a robot at work had on average 14% higher levels of RAW compared with those with no experience of working with robots. Furthermore, those who had experience with robots at home had on average 10% higher appraisal of robots in general. Healthcare professionals who reported having used a robot at home had on average 9% higher levels of RAW and 8% higher levels of GVR.

As another coherent result, respondents with managerial experience had more (4%–9%) positive views on robots. In the population sample, the employed respondents had on average 8% lower levels of RAW. Male gender predicted consistently higher (6%–10%) acceptance in the population, yet the healthcare professional data only showed higher RAW for males and no differences in GVR between genders. Younger age predicted higher levels of GVR and RAW in the population sample but lower GVR and RAW among the healthcare professionals.

## 4 DISCUSSION

We examined the experience healthcare professionals have with robots and how it associates with attitudes towards robots. The attitudes among the healthcare professionals were reflected to the attitudes in the population. We also analysed how robot assistance is approved depending on the care task in question. Hypotheses for healthcare data were based on nature of activities theory (Santoni de Sio & van Wynsberghe, 2016) and segmentation of care tasks (Ballermann et al., 2011). The two hypotheses regarded tasks for which healthcare professionals would find robot assistance most agreeable.

The results show that general views on robots are more positive among the Finnish population compared with the healthcare professionals. Among occupational groups, practical nurses stood out as having the most reserved attitudes towards robots. Hence, even if laborious (Erkkilä et al., 2016; Trydegård, 2012) and partly routine‐like (Sparrow & Sparrow, 2006) practical care work in particular could benefit from robotics, the workers did not see this solely as positive. That said, this finding reflects the worries regarding quality and ethics of care, which are related to responsiveness, humanity and attentiveness (Beedholm et al., 2015; Hofmann, 2013; Sharkey, 2014; Vallor, 2011; van Wynsberghe, 2013). Regardless of their relatively negative attitudes towards robots in general, the healthcare professionals approved the idea of robot assistance in some care tasks rather than in others.

Reflected against the nature of activities approach (Santoni de Sio & van Wynsberghe, 2016), direct patient care is most often a practice‐oriented activity that might be as important as the end result; thus, the goal is internal to the activity (Ballermann et al., 2011). Indirect patient care and other activities of care work could, on the contrary, be considered as goal‐directed tasks. In our first hypothesis, we presumed that healthcare professionals would approve robot assistance in indirect rather than in direct patient care tasks. However, there was no such dichotomy found between views regarding robot assistance in handling materials or patients (e.g. Alaiad & Zhou, 2014; Jenkins & Draper, 2015). The hypothesis must be rejected, given that excluding the task of moving a patient, other robot‐assisted tasks with high agreement (moving, sorting and shelving materials) are considered indirect and goal oriented. Also, the respondents were least comfortable with the idea of robots assisting with more holistic tasks (van Wynsberghe, 2013, p. 427), namely telepresence health checks, unhygienic work, transferring patients on a stretcher and planning care or medication, which in part favoured the hypothesis.

Supporting our second hypothesis, the healthcare professionals found robot assistance most suitable for ergonomically challenging work (moving patients or heavy objects) as well as for tasks outside of actual nursing work (sorting, shelving and delivering materials). The results portray some of the challenges in nursing work. Healthcare professionals see robot assistants as being worthwhile for physically demanding tasks (Erkkilä et al., 2016; Trydegård, 2012) but also as translators and assisting in threatening situations. Firstly, we conclude that the assistive devices for heavy lifting in healthcare do not meet the current needs to a satisfactory level. Robots hold promise as more adequate next‐generation assistive tools that are either easy to use or even autonomous. Secondly, healthcare professionals deal with non‐native Finnish speakers, both as patients and as colleagues and end up taking care of, for example, immigrants with whom they do not have any common language at all. Robots as mobile virtual assistants could help with translating or sign language. Thirdly, we associate the approval of robot assistance in threatening situations to the home or residential care workers’ worries regarding aggressive behaviour among residents and their relatives (Banerjee et al., 2012).

Our results are consistent with the studies implicating that robots are viewed more positively after personal experience (Heerink, 2011; Louie et al., 2014; Nomura et al., 2006). The experience healthcare professionals have with robots at home or work outside healthcare is associated with higher acceptance of robots, both in general and as assistants at work. The difference is that most of the population's experiences with robots originate from work, whereas most of the healthcare professionals’ experiences originate from home. This also explains the reason behind the more frequent robot use among the population. People working in an industrial field are more familiar with robots than people who work in service fields due to the robotization of industrial production. Robot use at home was more frequently reported among the healthcare professionals compared with the population, but this most likely refers to the temporal 2‐year difference in data collection. Household robots such as autonomic vacuum cleaners have become more common in recent years.

The attitudes of healthcare professionals seem to be notably influenced by experiences with robots, or due to the obvious heterogeneous causality; those with more positive views on robots strive for opportunities to use robots. In the population sample, young men who had experience with robots at work had the most positive overall attitudes towards robots. Among healthcare professionals, the older respondents who had experience with robots at home had the most positive overall attitudes. This perhaps implies the heightened user needs (van Wynsberghe, 2013) among older healthcare professionals. Along ageing, the need for assistive technology becomes more relevant and appealing.

The gender differences seem to reflect the fact that there traditionally has been a cultural divide between masculine technological and feminine social professions in Finland. The differing gender profiles between these fields are still visible. Women represent just one‐fifth of all graduations in higher technological education but two‐thirds of graduations in social and healthcare education (Finnish Statistics, 2015). Increasing use of robotic and other ICT appliances in care tasks and organizations may change this picture. Intensive development and deployment of innovations in the care sector may make the sector more attractive to technologically oriented males and, oppositely, call for more technological training of human‐oriented healthcare professionals. It has indeed been implied that robots reproduce concepts of social order, such as gendered notions of work (Boyer, 2004).

Managerial experience emerged as an important factor explaining the variance in robot acceptance, especially in the healthcare professional data. In addition, head nurses and other managers had relatively extensive experience with robots in healthcare work. This corresponds with prior studies showing that higher education and managerial experience go hand in hand with robot acceptance (Beedholm et al., 2015; de Graaf & Ben Allouch, 2013). Managers consider technology, at least partly, from an organization's perspective. Investing in modern technology can be seen useful to attract both potential customers and top employees. On the other hand, employees may have their concerns over how implementing new technology will affect the substance or amount of their work.

Inconsistency was found in occupational status of whether the respondent was working at the time of the study. The employed respondents in the population sample had more negative attitudes towards robots, contrary to the healthcare sample. The pessimism in the general working population can indicate common uncertainties regarding job losses caused by the future automatization (Manyika et al., 2013; Pajarinen & Rouvinen, 2014), while the optimism among healthcare professionals may illustrate the view of holistic and emotional care (e.g. Scammell et al., 2017) which cannot be automated or done by robots. A theoretical modelling suggests that the probability of computerizing in care tasks is relatively low (Frey & Osborne, 2013), but up to now, there has been no empirical evidence of the labour effects or the possibilities to increase productivity in care services due to robotization.

In general, assessing productivity is far less straightforward for services than for industrial production, for which inputs and outputs can be objectively identified. The units of services are difficult to define and the quality of services will not necessarily improve in a direct relationship with bigger inputs (Grönroos & Ojasalo, 2004). Robotization of care service to enhance productivity sounds purely dystopic for many people: Will future care homes be staffed by robots only? Would the older people have contact mainly with machines that take care of delivering food, cleaning, doing medical measurements and organizing social activities?

### 4.1 Ethics and validity of the study

The study complies with the regulations of the Finnish Advisory Board of Research Integrity and more broadly with the Declaration of Helsinki. All of the participants were informed about the aims of the study and they had the right to decline participation. Consent was requested at the beginning of the survey and the data handling was designed to ensure the participants’ anonymity. No ethical approval was needed.

In Finland, a high majority (90%) of nurses are unionized (Kilpeläinen, 2010). This and the random sampling conducted support the representative nature of the healthcare professional data although female and older respondents were slightly over‐represented compared with the population (Ailasmaa, 2014) in the combined sample. As a limitation of this study, RAW was measured differently in the population and healthcare professional samples; thus, they were only compared by descriptive means.

## 5 CONCLUSIONS

Several differences were found between the population and healthcare professionals; thus, it seems fruitful to study healthcare workers as a distinct robot user group. Moreover, the differences among different level healthcare professionals show the further importance of acknowledging different interest groups in studies of robot acceptance.

The most constant finding between the respondent groups was that individuals who have experience with robots, have more positive attitudes towards them. Healthcare professionals have fairly optimistic expectations towards robot assistance, but only with certain kinds of tasks. Instead of viewing robotization as a form of dehumanizing care (Scammell et al., 2017), robots may be used to assist caregivers by doing practical routine work, thus allowing the workers to concentrate on human‐centred tasks (Sparrow & Sparrow, 2006). These results together underline the importance of developing proactive workplace practices where different‐level employees are able to plan together the possible implementations of care robotics.

## CONFLICT OF INTEREST

No conflict of interest has been declared by the authors.
