# Supplementary material for: Finnish healthcare professionals' attitudes towards robots: Reflections on a population sample
Source: Nurs Open. 2018 Mar 23;5(3):300–9. doi: 10.1002/nop2.138 (PMC6056472; doi:10.1002/nop2.138)
Supplement: Supplementary file 1 [file NOP2-5-300-s001.docx]

APPENDIX A

**In the last section of the questionnaire are examples of care work tasks, which could, in principle, be done with robots or done with robot assistance. Please rate every scenario by how comfortable you would feel about a robot assisting you with that specific task.**

1. Robot documenting patient information
2. Robotic and autonomous stretcher
3. Robot as a courier
4. Robot sorting and shelving goods
5. Robot as an interpreter (including sign language)
6. Telepresence robot in communication between patient and nursing staff, especially in emergency situations (picture below)
7. Telepresence robot in minor health checks
8. Robot planning care procedures (i.e., controlling medication interaction)
9. Robot assisting in unhygienic tasks
10. Robot moving heavy materials or large amount of goods
11. Robot assisting in moving or lifting a patient
12. Robo-powered suit (exoskeleton) for a care worker to wear while moving or lifting a patient
13. Robot assisting in threatening situations

**
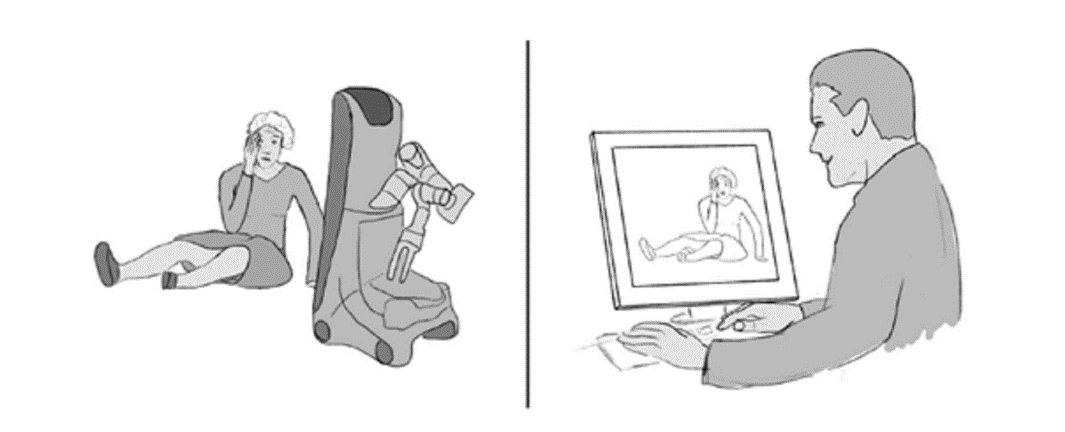
**

APPENDIX B

| **Correlations between healthcare professionals’ general view of robots, robot acceptance at work, gender, age, managerial status and overall experiences with robots** | | | | | |  |
| --- | --- | --- | --- | --- | --- | --- |
|  | 1. | 2. | 3. | 4. | 5. |  |
| 1. General view of robots |  |  |  |  |  |  |
| 2. Robot acceptance at work | .605^**^ |  |  |  |  |  |
| 3. Male | .090^**^ | .004 |  |  |  |  |
| 4. Age | .044^*^ | .063^**^ | -.041^*^ |  |  |  |
| 5. Managerial status | .101^**^ | .092^**^ | .054^**^ | .182^**^ |  |  |
| 6. Robot experiences | .152^**^ | .101^**^ | .073^**^ | -.009 | .044^*^ |  |
|  |  |  |  |  |  |  |
| ***. Correlation is significant at the 0.01 level (2-tailed).* | | | | | |  |
| **. Correlation is significant at the 0.05 level (2-tailed).* | | | | | |  |
